# Characterizing symptom burden in knee osteoarthritis: the predominant role of Kellgren-Lawrence grade 4 in functional decline and pain severity

**DOI:** 10.1007/s00296-026-06136-x

**Published:** 2026-06-03

**Authors:** Sibel Bozgeyik-Bagdatli, Sema Nur Aslan, Ömür Çağlar, Gizem İrem Kınıklı

**Affiliations:** 1 Department of Musculoskeletal Physiotherapy and Rehabilitation, Faculty of Physical Therapy and Rehabilitation, Hacettepe University, Samanpazari, 06100 Ankara, Turkey; 2 Department of Physiotherapy and Rehabilitation, Faculty of Health Sciences, Atılım University, Kızılcaşar Neighborhood, Incek, Gölbaşı, 06836 Ankara, Turkey; 3 Department of Orthopedics and Traumatology, Faculty of Medicine, Hacettepe University, 06100 Ankara, Turkey

**Keywords:** Activities of daily living, Muscle strength, Osteoarthritis, Knee, Pain measurement, Physical functional performance

## Abstract

Knee osteoarthritis (KOA) causes pain, functional impairments, and sleep disturbances. This study aimed to assess differences in pain pattern, insomnia, function, and muscle strength across Kellgren–Lawrence grades in patients with KOA. In this cross-sectional study, 82 patients (65.8 ± 7.8 years) were categorized into Kellgren–Lawrence Grade 2 (*n* = 20), Grade 3 (*n* = 20), and Grade 4 (*n* = 42). Pain (VAS, ICOAP), insomnia (ISI), knee function (KOS-ADLS), functional performance (Timed-Up and Go [TUG], Five Times Sit-to-Stand [FTSST]), and isometric muscle strength were evaluated. Comparisons were performed using ANCOVA, adjusting for age and BMI. Groups differed significantly in age and BMI (*p* < 0.001 and *p* = 0.024), with higher values in Grade 4. Significant between-group differences were observed in VAS (*p* = 0.006), ICOAP-constant pain (*p* = 0.037), ICOAP-total pain (*p* = 0.035), TUG (*p* = 0.021), KOS-ADLS (*p* = 0.015), and quadriceps strength of the affected knee (*p* < 0.001) and hip adductor strength (*p* = 0.046). No significant differences were found for ICOAP-intermittent pain, ISI, or non-affected knee quadriceps strength (*p* > 0.05). Pairwise comparisons showed that Grade 4 patients had significantly worse outcomes than Grade 2 in VAS (*p* = 0.013), ICOAP-constant pain (*p* = 0.041), and total pain (*p* = 0.047), and TUG (*p* = 0.025), as well as reduced quadriceps strength of both affected (*p* < 0.001) and non-affected (*p* = 0.019) knees. Differences between Grades 2 and 3 were limited to VAS (*p* = 0.026), while Grades 3 and 4 differed only in KOS-ADLS (*p* = 0.022). Higher Kellgren–Lawrence grades were associated with greater pain, poorer function, and reduced quadriceps strength, particularly in Grade 4, emphasizing the clinical relevance of radiographic grading in characterizing symptom burden in KOA.

**Supplementary Information:**

The online version contains supplementary material available at 10.1007/s00296-026-06136-x.

## Introduction

Osteoarthritis (OA) is currently defined as a multifactorial disorder involving not only the intraarticular cartilage but also the subchondral bone, periarticular soft tissues, and the entire joint complex [1]. Knee osteoarthritis (KOA) is one of the most common types of osteoarthritis and usually characterized by pain and loss of function, which lead to disability [2]. According to estimates from the Global Burden of Disease Study in 2017, the global prevalence of KOA was approximately 300 million, highlighting the substantial burden of the disease [3].

Although various diagnostic options are available, radiographic imaging remains the most used method for diagnosing KOA [4]. In radiographic diagnosis, the Kellgren-Lawrence (KL) classification is commonly used for classifying OA severity. The KL grades are classified as follows: Grade 0 (no radiographic feature), Grade 1 (doubtful joint space narrowing), Grade 2 (definite osteophytes and possible joint space narrowing), Grade 3 (multiple osteophytes, definite joint space narrowing, sclerosis, possible bone deformity) and Grade 4 (large osteophytes, marked joint space narrowing, severe sclerosis and definite bone deformity) [5].

KOA may negatively affect functional status, pain severity, pain pattern (intermittent, constant, and mixed), and muscle strength [6, 7]. Moreover, sleep disturbances, particularly insomnia, are frequently reported among older adults with KOA, potentially due to pain-related discomfort and reduced physical function [8]. However, studies investigating the association between clinical symptoms and the radiographic severity of KOA have yielded conflicting results, as patients often present symptoms that do not align with their radiological findings [4].

For instance, while some studies have demonstrated a strong relationship between radiologic OA and pain severity [7, 9], or identified muscle strength as a determinant of radiologic OA severity [6], others have reported contrasting results. Notably, Bastick et al. reported no association between quadriceps muscle strength and radiographic KOA severity, while identifying knee pain as a more relevant clinical indicator [10].

Given these inconsistencies, the present study aimed to clarify the relationship between KOA symptoms and radiographic severity by examining whether pain intensity, pain pattern, insomnia, knee-related function, functional performance, and isometric muscle strength differ across KL grades.

## Methods

### Study design

This cross-sectional study was conducted between March 2024 and March 2025 at Hacettepe University Hospital Department of Musculoskeletal Physiotherapy and Rehabilitation. The study was carried out according to the guidelines of the Declaration of Helsinki (October 2024 version). Ethical approval for the study was obtained from the Hacettepe University Institutional Ethics Committee of Non-Interventional Studies (approval number 2024/07–25, protocol number SBA 24/460 Date: 02.04.2024). Informed consent was obtained from all patients. The study has been written according to the international recommendations for Strengthening the Reporting of Observational Studies in Epidemiology (STROBE) [11].

### Participants

A total of 82 patients diagnosed with unilateral KOA, age between 45 and 80 years old were recruited for the study. Inclusion criteria were as follows: patients aged between 45 and 80 years, diagnosis of unilateral knee osteoarthritis confirmed by clinical and radiological findings, radiographic KOA graded as 2 to 4 according to the Kellgren–Lawrence classification, presence of knee pain for at least 3 months; and independent ambulation without assistive devices. Radiographic evaluations were performed and interpreted by the Department of Radiology. Patients were referred to our clinic with established diagnoses and K-L grades based on standardized weight-bearing anteroposterior knee radiographs. Patients were excluded from the study if they had the presence of any neurological, rheumatological, and/or oncological conditions, a history of knee or hip surgery, had any intra-articular injection within the past 6 months, severe cardiopulmonary disease limiting functional capacity; or concomitant lower extremity musculoskeletal disorders affecting gait or functional performance and had any cognitive impairment that prevents understanding the questionnaire items. Patients were categorized into groups according to their radiographic classification: those with grade 2 KOA were assigned to Group 2, grade 3 KOA to Group 3, and grade 4 KOA to Group 4.

### Sample size calculation

A post hoc power analysis was performed using G*Power version 3.1 for an ANCOVA model (fixed effects, main effects and interactions) with three groups and two covariates (age and BMI), based on a total sample size of 82 participants and an alpha level of 0.05. Using the observed effect size derived from the primary outcome (pain intensity) (Cohen’s f = 0.38), the achieved statistical power was calculated as 0.87. These findings indicate that the present sample size was sufficiently powered to detect medium-to-large to large effects for the primary outcome. The post hoc power analysis was conducted based on the observed effect sizes and sample size; however, this analysis was considered descriptive only.

### Outcomes

#### Demographic characteristics

Demographic and clinical characteristics including age, sex, body weight, height, body mass index (BMI), radiological grade, more affected knee and dominant lower limb were recorded. The dominant lower limb was determined by asking the participants which foot they would prefer to use to kick a ball rolling toward them.

#### Pain intensity assessment

The primary outcome measure of the study was the pain intensity. Current knee pain intensity was measured using the Visual Analog Scale (VAS). VAS is widely accepted valid and reliable tool for assessing pain intensity in patients with KOA. Patients were asked to mark their current knee pain intensity on a 100 mm horizontal line where 0 (starting point) indicates ‘no pain’ and 100 (end-point) indicates ‘the worst imaginable pain’ [12].

#### Pain pattern

Pain pattern (intermittent, constant, or mixed) was assessed using the measure of Intermittent and Constant Osteoarthritis Pain (ICOAP). The ICOAP is an 11-item scale recommended by OARSI/OMERACT for evaluating pain patterns in patients aged 40 years and older with OA. It consists of 2 subscales: one assessing constant pain and the other assessing intermittent pain (pain that comes and goes), both based on the experience of pain over the past week. Items scored using a 5-point Likert scale, where 0 indicates ‘not at all’ and 4 indicates ‘very often’. Higher scores indicate more severe pain [13].

#### Insomnia assessment

Insomnia was assessed with the Insomnia Severity Index (ISI), a validated 7-item questionnaire designed to evaluate the severity and impact of insomnia [14]. Each item is scored on a 5-point Likert scale, where 0 indicates ‘not at all’ and 4 indicates ‘extremely’, with a total score ranging from 0 to 28, where higher scores indicate more severe insomnia.

#### Knee-related function assessment

Knee function was evaluated using the Knee Outcome Survey–Activities of Daily Living Scale (KOS-ADLS), comprising 14 items: six on symptoms (pain, stiffness, swelling, giving way, weakness, limping) and eight on functional activities (walking, stair use, standing, kneeling, squatting, sitting, rising from a chair). Items are rated on a 5-point Likert scale (0 = most severe, 5 = no symptom/limitation), with higher total scores indicating better knee function [15].

#### Functional performance assessment

Five times sit-to-stand test (FTSST) and Timed Up and Go (TUG) test were used to assess functional performance. Functional tests were conducted using a standard test chair (seat height 48 cm; arm height 68 cm). For the FTSST patients asked to cross their arms over their chest. Upon the physiotherapist’s command to ‘start’, patients are instructed to stand up and sit down five times as quickly and safely as possible without using their arms for assistance. The total time required to complete the five times is measured using a stopwatch and recorded in seconds [16]. For the TUG, the patients are instructed to rise from a standard chair, walk 3 m as quickly and safely as possible, turn around, and return to sit on the chair [17]. All functional performance tests were administered by a single experienced evaluator to ensure consistency and minimize inter-rater variability.

#### Isometric muscle strength assessment

Quadriceps and hip adductor strength were assessed using the K-Bubble device (Kinvent, Montpellier, France), a portable, wireless pneumatic dynamometer compatible with valve-equipped pressure units or balls of various sizes, providing real-time biofeedback via a digital application (mmHg). Muscle strength was evaluated during maximum voluntary isometric contractions (MVIC) using the “make test,” in which participants exert maximal force against the fixed device for standardized and reproducible quantification of isometric performance [18].

**Fig. 1:**
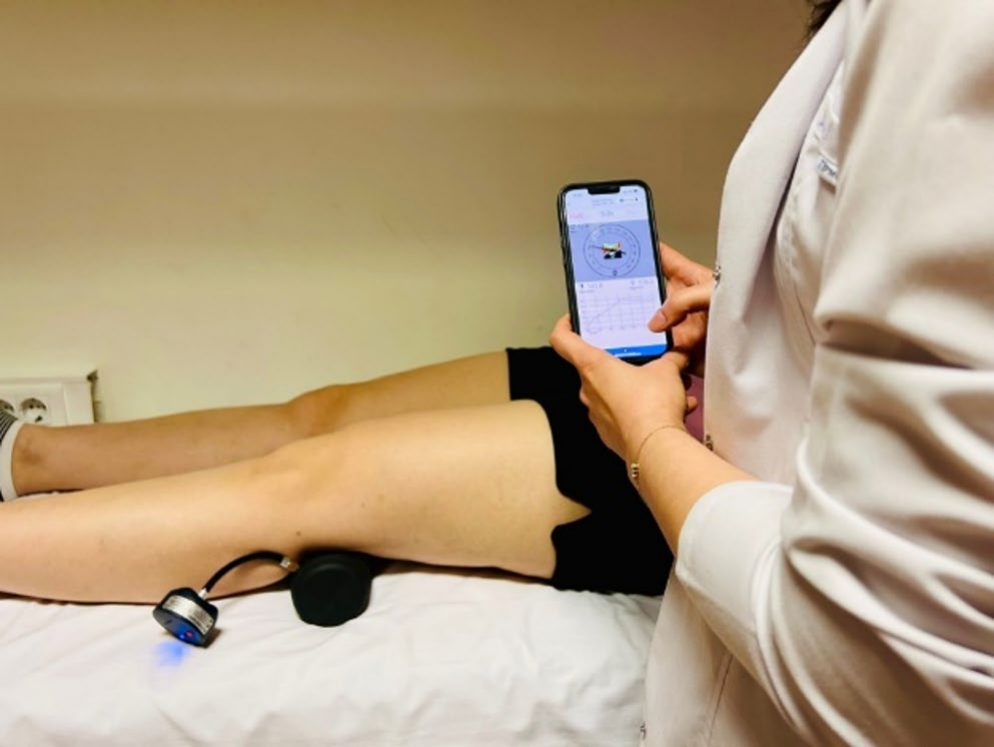
Isometric quadriceps muscle strength assessment

For quadriceps assessment, patients were positioned in long sitting, with the device placed behind the knee. They performed MVIC knee extension by pressing the ball against the bed (Fig. 1). Hip adductor strength was measured in a seated position with the device between the knees, performing bilateral MVIC adduction by squeezing the ball [18, 19]. Each test was repeated three times, and the highest value was recorded digitally in kilograms. All muscle strength assessments were also conducted by a single experienced physiotherapist using a standardized protocol to reduce inter-rater variability.

### Statistical analysis

Statistical analyses were performed using the SPSS software (ver. 26.0, SPSS Inc, Chicago, IL). The normality assumption was assessed using both analytical (Kolmogorov-Smirnov Test, skewness, and kurtosis) and visual methods (histograms and detrended plots). Descriptive statistics were presented as median (minimum–maximum) for non-normally distributed data and as mean ± standard deviation for normally distributed data. For normally distributed continuous variables, group comparisons were performed using one-way analysis of variance (ANOVA). Categorical variables were analyzed using Fisher’s exact test or Pearson’s chi-square test, as appropriate. For between-subjects’ analyses, analysis of covariance (ANCOVA) was performed to adjust for potential confounding variables, including age and BMI. Effect sizes for the ANCOVA models were reported as partial eta squared (η²p). To control for Type I error, post hoc pairwise comparisons were conducted using Bonferroni correction. A p-value of less than 0.05 was considered statistically significant.

## Results

The analysis included 82 patients who had KOA (Grade 2 n: 20, Grade 3 n:20, Grade 4 n: 42) and the mean age of the patients was 65.83 ± 7.79 years. No significant difference was found between the groups in terms of weight, height, and BMI (*p* = 0.118; *p* = 0.459 respectively), while significant difference was found between the groups in terms of age and BMI (*p* < 0.001; *p* = 0.024 respectively). Comparison of the demographic characteristics of the patients are shown in Table 1. Post-hoc test for multiple comparisons showed that patients who had Grade 4 KOA were older (*p* < 0.001) and had a higher BMI (*p* = 0.019).

**Table 1:** Demographic characteristics comparisons of the groups

ANOVA		Grade 2 KOA (*N* = 20)	Grade 3 KOA (*N* = 20)	Grade 4 KOA (*N* = 42)		
		X ± SD			F_(df)_	*p*
**Age (y)**		62.65 ± 6.95	60.40 ± 7.95	69.93 ± 5.63	17.285_(2)_	< **0.001**
**Weight (kg)**		74.70 ± 12.57	79.90 ± 13.05	81.64 ± 11.63	2.199_(2)_	0.118
**Height (m)**		1.62 ± 0.07	1.62 ± 0.06	1.59 ± 0.08	0.786_(2)_	0.459
**BMI (kg/m** ^2^ **)**		28.45 ± 4.52	30.34 ± 4.86	32.04 ± 4.80	3.915_(2)_	**0.024**

Chi-square		N frequency (%)			Chi sqaure	p
**Gender**	**Female**	17 (20.7%)	18 (22.0%)	38 (46.3%)	0.638	*0.895
	**Male**	3 (3.7%)	2 (2.4%)	4 (4.9%)		
**Affected knee**	**Right**	12 (14.6%)	10 (12.2%)	21 (25.6%)	0.606	**0.738
	**Left**	8 (9.8%)	10 (12.2%)	21 (25.6%)		
**Dominant knee**	**Right**	18 (22.0%)	18 (22.0%)	40 (48.8%)	0.634	*0.773
	**Left**	2 (2.4%)	2 (2.4%)	2 (2.4%)		

*G2* Kellgren-Lawrence Grade 2 osteoarthritis, *G3* Kellgren-Lawrence Grade 3 osteoarthritis, *G4* Kellgren-Lawrence Grade 4 osteoarthritis, *KOA* Knee Osteoarthritis, *BMI* Body Mass Index, *X ± SD* Mean ± Standard Deviation, *F* ANOVA test statistics, *df* Degree of freedom, *p* < 0.05 means statistical significance

*Fisher’s exact test, **Pearson’s chi-square test

Comparisons of patients’ outcomes were performed between three groups according to Kellgren–Lawrence grades (Grade 2 to 4) after adjustment for age and BMI. Significant between-group differences were found in VAS (*p* = 0.006, η²*p* = 0.125), ICOAP-constant pain score (*p* = 0.037, η²*p* = 0.084), ICOAP-total pain score (*p* = 0.035, η²*p* = 0.086), TUG (*p* = 0.021, η²*p* = 0.096), KOS-ADLS (*p* = 0.015, η²*p* = 0.105), affected knee quadriceps muscle strength (*p* < 0.001, η²*p* = 0.229), and hip adductor strength (*p* = 0.046, η²*p* = 0.083). Between-group comparisons are presented in Table 2.

**Table 2:** Between-group analysis after adjustment for age and BMI

Outcomes		Group	X ± SD	F_(2,77)_	η²*p*	*p* Value
**VAS**		G-2 G-3 G-4	4.45 ± 1.93 6.20 ± 2.14 6.30 ± 1.98	5.431	0.125	**0.006**
**ICOAP**	**Constant pain score**	G-2 G-3 G-4	2.65 ± 5.71 6.50 ± 7.28 7.80 ± 7.23	3.457	0.084	**0.037**
	**Intermittent pain score**	G-2 G-3 G-4	12.30 ± 5.37 15.25 ± 7.41 15.63 ± 6.74	1.176	0.030	0.314
	**Total pain score**	G-2 G-3 G-4	14.95 ± 9.96 21.30 ± 10.27 23.42 ± 10.85	3.513	0.086	**0.035**
**ISI**		G-2 G-3 G-4	7.20 ± 6.72 12.75 ± 7.54 10.45 ± 8.32	2.822	0.070	0.066
**FTSST**		G-2 G-3 G-4	13.25 ± 3.17 14.65 ± 5.50 20.63 ± 12.20	1.375	0.035	0.259
**TUG**		G-2 G-3 G-4	8.68 ± 2.09 9.21 ± 1.84 16.45 ± 8.97	4.051	0.096	**0.021**
**KOS-ADLS**		G-2 G-3 G-4	47.64 ± 22.14 49.57 ± 21.50 35.92 ± 12.39	4.469	0.105	**0.015**
**Quadriceps muscle strength**	**Non-affected knee**	G-2 G-3 G-4	24.36 ± 8.37 20.94 ± 4.93 19.18 ± 5.87	2.468	0.063	0.092
	**Affected knee**	G-2 G-3 G-4	25.71 ± 7.58 20.26 ± 3.70 16.87 ± 5.49	10.835	0.229	< **0.001**
**Hip adductor strength**		G-2 G-3 G-4	31.83 ± 12.82 25.90 ± 8.97 23.20 ± 11.48	3.227	0.083	**0.046**

*VAS* Visual analog scale, *FTSST* Five time sit to stand test, *TUG* Timed up and go test, *ICOAP* Intermittent and Constant Osteoarthritis Pain Scale, *ISI* Insomnia Severity Index, *KOS-ADLS* Knee Outcome Survey-Activities of Daily Living Scale, *G-2* Kellgren-Lawrence Grade 2 osteoarthritis, *G-3* Kellgren-Lawrence Grade 3 osteoarthritis, *G-4* Kellgren-Lawrence Grade 4 osteoarthritis, X ± SD Mean±standard deviation, F_(2,77)_ F statistic with corresponding numerator and denominator degrees of freedom derived from the analysis of covariance, η²p Partial eta squared, ANCOVA, *p* < 0.05 means statistical significance

Pairwise comparisons were performed using post hoc tests with Bonferroni adjustment for outcomes that showed significant differences between groups (VAS, ICOAP-constant pain score, ICOAP-total score, TUG, KOS-ADLS, quadriceps muscle strength of the affected knee, and hip adductor strength) (Table 3). Significant differences were observed between Grade 2 and Grade 4 in VAS (*p* = 0.013), ICOAP-constant pain score (*p* = 0.041), ICOAP-total score (*p* = 0.047), TUG (*p* = 0.025), and KOS-ADLS (*p* = 0.055, borderline significance), while quadriceps muscle strength of the affected knee did not reach statistical significance in this comparison (*p* < 0.001 indicates strong significance depending on direction) (Table 3).

**Table 3:** Pairwise comparisons

			Pairs	Mean difference	SE	p-Value	95% CI
**VAS**			G2-G3	– 1.761	0.653	**0.026**	[– 3.359, – 0.164]
			G2-G4	– 1.938	0.656	**0.013**	[– 3.545, – 0.331]
			G3-G4	– 0.177	0.668	1.000	[– 1.813, 1.459]
**ICOAP**		**Constant pain score**	G2-G3	– 4.116	2.234	0.208	[– 9.586, 1.354]
			G2-G4	– 5.691	2.251	**0.041**	[– 11.203, – 0.179]
			G3-G4	– 1.575	2.291	1.000	[– 7.186, 4.035]
		**Total score**	G2-G3	– 6.791	3.367	0.142	[– 15.036, 1.453]
			G2-G4	– 8.396	3.393	**0.047**	[– 16.704, – 0.088]
			G3-G4	– 1.605	3.453	1.000	[– 10.061, 6.852]
**TUG**			G2-G3	– 1.124	2.045	1.000	[– 6.131, 3.882]
			G2-G4	– 5.585	2.057	**0.025**	[– 10.621, – 0.550]
			G3-G4	– 4.461	2.094	0.109	[– 9.588, 0.666]
**KOS-ADLS**			G2-G3	– 2.278	5.705	1.000	[– 16.245, 11.688]
			G2-G4	13.831	5.738	0.055	[– 0.217, 27.879]
			G3-G4	16.109	5.842	**0.022**	[1.806, 30.412]
**Muscle strength**	**Quadriceps (affected knee)**		G2-G3	5.627	1.881	**0.011**	[1.018, 10.235]
			G2-G4	8.667	1.896	< **0.001**	[4.020, 13.313]
			G3-G4	3.040	1.915	0.350	[– 1.653, 7.733]
	**Hip adductors**		G2-G3	6.978	3.703	0.191	[– 2.102, 16.057]
			G2-G4	8.987	3.722	0.055	[– 0.140, 18.113]
			G3-G4	2.009	3.726	1.000	[– 7.126, 11.144]

*VAS* Visual analog scale, *FTSST* Five time sit to stand test, *TUG* Timed up and go test, *ICOAP* Intermittent and Constant Osteoarthritis Pain Scale, *KOS-ADLS* Knee Outcome Survey-Activities of Daily Living Scale, *G2* Kellgren-Lawrence Grade 2 osteoarthritis, *G3* Kellgren-Lawrence Grade 3 osteoarthritis, *G4* Kellgren-Lawrence Grade 4 osteoarthritis, *SE* Standard Error, *CI* Confidence Interval, Post-hoc test with Bonferroni correction following ANCOVA, *p* < 0.05 means statistical significance

Between Grade 2 and Grade 3, a significant difference was found only in VAS (*p* = 0.026). Additionally, a significant difference between Grade 3 and Grade 4 was observed only for KOS-ADLS (*p* = 0.022), whereas no significant differences were found for ICOAP-constant pain score, ICOAP-total score, TUG, quadriceps strength, or hip adductor strength (*p* > 0.05). No other pairwise comparisons reached statistical significance (*p* > 0.05) (Table 3).

## Discussion

This study investigated differences in pain intensity, pain pattern, insomnia, knee-related function, functional performance, and isometric muscle strength among patients with KOA across radiographic severity levels defined by the KL classification. While no significant differences were found between groups in terms of weight and height, patients with KL Grade 4 were significantly older and had higher BMI values compared to the other groups.

Overall, increasing radiographic severity was associated with a marked deterioration in several clinical outcomes. After adjustment for age and BMI, significant between-group differences were observed in pain intensity, functional performance, constant and total pain, knee-related activities of daily living, and isometric quadriceps strength of the affected knee, and hip adductor strength. In contrast, intermittent pain, insomnia severity, and quadriceps strength of the non-affected knee did not differ significantly across groups.

Pairwise comparisons indicated that these differences were most pronounced between KL Grade 2 and Grade 4. Specifically, significant differences between these groups were observed in pain intensity, constant and total pain, functional performance, knee-related activities of daily living, and quadriceps strength. Pain intensity differed between Grades 2 and 3, whereas knee-related activities of daily living differed between Grades 3 and 4. Collectively, these findings suggest that greater radiographic severity is associated with worse clinical status; however, the progression of symptoms and functional impairments does not appear to follow a strictly linear pattern across disease stages.

### Pain and pain pattern

There is no consensus in the literature regarding changes in pain patterns across KL grades in patients with KOA, mostly due to the different pain mechanisms underlying intermittent, constant, or mixed pain patterns [20, 21]. Carlesso et al., reported that greater radiographic severity was associated with mixed-type pain (constant plus intermittent) [20]. In agreement with this findings, our results demonstrated that constant and total pain scores were significantly higher in KL grade 4 compared to grade 2, whereas intermittent pain did not differ significantly among KL grades 2, 3, and 4. The lack of significant differences adjacent grades (2–3 and 3–4) may reflect heterogeneity within KL categories, where radiographic classification does not fully capture the complexity of symptom presentation. Moreover, pain severity may be influenced by factors beyond structural changes visible on radiographs, such as synovitis or other peripheral and central pain mechanisms [22]. Supporting this perspective, Liu et al., in line with Philpott et al., demonstrated that the presence of an unacceptable symptom state was associated with greater severity of both intermittent and constant pain in individuals with KOA [23].

Another focus of this study was pain severity across KL grades. Our findings demonstrated that pain severity was significantly higher in KL grades 3 and 4 compared to grade 2, indicating an association between higher radiographic severity and greater pain severity. These findings are consistent with previous studies reporting that higher radiological severity is generally associated with greater pain severity, whereas persistent knee pain is less common in earlier stages of the disease [7, 24]. Importantly, no significant difference in pain severity was observed between KL grades 3 and 4. This may suggest that at more advanced radiographic stages differences in structural severity are not necessarily accompanied by proportional differences in perceived pain. This observation supports the notion that the relationship between structural damage and pain may not be strictly linear.

Importantly, although pain severity was comparable between grades 3 and 4, differences in pain patterns were still evident across KL grades. This underscores the need to evaluate pain not only in terms of intensity but also in terms of its qualitative characteristics. Our findings suggest that radiographic severity is associated with how pain is experienced and expressed. Therefore, clinical assessment and rehabilitation strategies should consider both pain severity and pain pattern to support more individualized management of patients with KOA.

### Insomnia

Insomnia and sleep disturbances are common in patients with KOA and have been associated with both pain and radiographic severity in previous studies [25–27]. In the present study, insomnia was assessed using the Insomnia Severity Index with established cut-off values defining ≤ 7 as no insomnia, 8–14 as sub-threshold insomnia, and 15–28 as clinical insomnia [28], According to these thresholds, patients with grade 2 KOA had a median ISI score of 6 (no insomnia), while those with grade 3 and grade 4 KOA had median scores of 11.5 and 10, indicating sub-threshold insomnia. Although this distribution suggests a tendency toward greater insomnia severity with increasing radiographic stage, the differences did not reach statistical significance. This finding may be explained by the multifactorial nature of sleep disturbances in KOAThis finding may be explained by the multifactorial nature of sleep disturbances in KOA [29, 30]. While higher KL grades have been linked to poorer sleep quality, this relationship is often mediated by pain—particularly nocturnal pain—rather than reflecting a direct effect of structural severity [31]. In our cohort, although pain intensity differed between groups, insomnia scores did not follow a similar pattern, supporting the concept of discordance between radiographic findings and patient-reported outcomes. Overall, these results suggest that insomnia in KOA is not solely dependent on radiographic severity but reflects a complex interaction of clinical and psychosocial factors. It should also be noted that the cross-sectional nature of the study and the use of a global insomnia measure may have limited the detection of subtle, stage-related differences in sleep disturbances.

### Knee-related function and functional performance

In the literature the association between physical function, functional performance and radiographic severity in KOA remains controversial [32, 33]. Sonobe et al. reported that radiographic severity was associated with decreased function only in patients with KL grade 4 and suggested that radiographic severity alone may be insufficient to fully explain functional impairment [32]. In line with Sonobe et al.’s findings, significant differences in TUG time were observed between KL grades 2 and 4 and between KL grades 3 and 4, whereas no significant difference was found between grades 2 and 3 in our study. Nevertheless, Özden et al. demonstrated that increasing radiographic severity was associated with poorer functional performance measured by the TUG test while no significant association was found with patient-reported outcomes, supporting the idea that performance-based measures may better reflect structural disease progression [34]. Moreover, Pereira et al. reported that increasing radiographic severity in knee osteoarthritis was associated with worsening physical function [35]. In addition to the TUG test, the FTSST was used to evaluate functional performance, however, FTSST scores did not differ significantly across KL grades. This discrepancy may suggest that different functional performance measures reflect slightly different aspects of physical function, and that TUG and FTSST may not demonstrate parallel changes across radiographic severity in our cohort. Together, these findings suggest that worse functional performance is associated with higher radiographic severity, particularly in individuals classified as KL grade 4, while functional performance appears comparable between KL grades 2 and 3. Supporting this interpretation, Liikavainio et al. also reported no significant association between radiographic severity and functional outcomes in men with knee osteoarthritis, although patients with more advanced radiographic grades tended to demonstrate poorer performance in functional tasks [33].

Physical function in activities of daily living and symptom severity were evaluated using the KOS-ADLS, with a significant difference observed only between KL grades 3 and 4. In line with our study, previous studies have reported that greater radiographic severity is associated with increased functional limitations, particularly in individuals with KL grade 4 KOA [6, 34–38]. While our findings align with this evidence, an important contribution of the present study is the use of both FTSST and KOS-ADLS to provide a more comprehensive comparison of functional status across KL grades.

### Muscle strength

Weakness of the knee extensors has been identified as an important risk factor associated with radiographic KOA severity, and quadriceps weakness is frequently observed in patients with more severe KOA [6, 39, 40]. Furthermore, there are studies in the literature reporting decreased hip adductor strength in patients with KOA [41]. A previous systematic review has suggested that the decline in muscle strength in individuals with knee OA cannot be explained solely by reductions in lean muscle mass [42]. Although direct comparisons of muscle strength across KL grades are limited, our findings demonstrated significantly lower quadriceps strength in individuals with higher radiographic severity. Specifically, quadriceps strength of the affected knee differed significantly between KL Grades 2 and 4 and between Grades 2 and 3. These results suggest that progressive radiographic joint degeneration may contribute to muscle weakness in knee OA, while higher radiographic severity appears to be associated with lower quadriceps strength. No significant pairwise differences were found in hip adductor strength across KL grades, despite a significant overall effect in the ANCOVA model. The discrepancy between overall and pairwise results for hip adductor strength may suggest a more subtle or distributed effect across groups rather than distinct between-group differences. Although pairwise comparisons did not demonstrate statistically significant differences, hip adductor strength may still be clinically relevant when considered in relation to functional performance and the distribution of values across severity groups.

### Limitations

This study has several limitations. First, it was conducted at a single center, which may limit the generalizability of the findings. Therefore, future research involving larger, multicenter cohorts is recommended to enhance external validity. Second, muscle strength measurements may have been influenced by pain during testing, which could lead to an underestimation of true maximal voluntary force due to pain-related arthrogenic muscle inhibition. Another important limitation of this study is its cross-sectional design, which does not allow evaluation of temporal changes in muscle weakness and functional decline across different stages of knee osteoarthritis. Accordingly, it is not possible to determine whether these impairments are primary contributors to disease progression or secondary consequences of osteoarthritis severity. Due to its cross-sectional nature, the study also does not permit causal inferences between radiographic severity and pain, functional status, or muscle strength outcomes. Therefore, the findings should be interpreted as associations rather than causal relationships across Kellgren–Lawrence grades. The study included only patients with Kellgren–Lawrence grades 2–4, as early-stage osteoarthritis (KL 0–1) patients rarely present to physiotherapy services due to minimal symptom severity. Therefore, the findings should not be generalized to early-stage knee osteoarthritis populations. The unequal distribution of sample sizes across Kellgren–Lawrence grades represent a limitation of this study, as it reflects the natural clinical population but may have influenced the statistical comparisons between groups. In addition, significant differences in age and body mass index were observed between KL grades, with patients in Grade 4 being older and having higher BMI values. These variables may have acted as potential confounders influencing pain, muscle strength, and functional performance outcomes. Although height and body weight were comparable across groups, the higher age and BMI in advanced stages may partially explain the observed between-group differences. Therefore, the results should be interpreted with caution, and future studies incorporating age- and BMI-adjusted analyses are warranted.

## Conclusion

In conclusion, the present findings suggest that advanced radiographic severity, particularly Kellgren–Lawrence grade 4, is associated with higher reported pain levels and poorer functional outcomes, including reduced knee-related function, functional performance and quadriceps strength. Differences observed across severity levels appear to be largely driven by individuals in the most advanced stage of the disease. These results highlight a meaningful association between structural disease severity and clinical presentation and support the value of incorporating multidimensional assessments of pain function, and functional performance in the clinical evaluation of individuals with KOA. While early identification and targeted management of these impairments may be clinically relevant, the cross-sectional design of this study precludes any causal inferences or conclusions regarding disease progression.

## Electronic Supplementary Material

Below is the link to the electronic supplementary material.

Supplementary Material 1

## Data Availability

Raw data are available upon reasonable request from the corresponding author.
