# Supplementary material for: Characterizing symptom burden in knee osteoarthritis: the predominant role of Kellgren-Lawrence grade 4 in functional decline and pain severity
Source: Rheumatol Int. 2026 Jun 3;46(6):126. doi: 10.1007/s00296-026-06136-x (PMC13233923; doi:10.1007/s00296-026-06136-x)
Supplement: Supplementary file 1 — Supplementary Material 1 [file 296_2026_6136_MOESM1_ESM.docx]

**TABLES**

| Supplementary Material 1. Comparison of outcomes between groups | | | | | | |
| --- | --- | --- | --- | --- | --- | --- |
| Outcomes | | Group | Median (IQR) | Mean of the ranks | X^2(2)^ | p-Value |
| VAS | | G-2  G-3  G-4 | 3.85 (2.50)  6.10 (2.78)  6.00 (3.53) | 25.63  46.43  46.71 | 11.784 | **0.003** |
| ICOAP | **Constant pain score** | G-2  G-3  G-4 | 0.00 (0.00)  3.50 (13.75)  9.00 (14.50) | 30.05  42.20  45.76 | 7.137 | **0.028** |
|  | **Intermittent pain score** | G-2  G-3  G-4 | 12.00 (6.75)  16.00 (11.00)  18.00 (6.50) | 30.35  43.70  44.88 | 5.495 | 0.064 |
|  | **Total pain score** | G-2  G-3  G-4 | 12.50 (9.75)  20.00 (13.75)  23.00 (15.50) | 26.40  42.23  47.52 | 10.932 | **0.004** |
| ISI | | G-2  G-3  G-4 | 6.00 (10.25)  11.50 (11.25)  10.00 (11.50) | 31.80  49.15  41.51 | 5.496 | 0.064 |
| FTSST | | G-2  G-3  G-4 | 12.98 (3.63)  12.53 (5.46)  16.79 (10.87) | 30.88  33.90  50.18 | 11.597 | **0.003** |
| TUG | | G-2  G-3  G-4 | 8.44 (1.83)  9.06 (3.70)  14.40 (8.14) | 23.38  29.35  55.92 | 32.187 | **< 0.001** |
| KOS-ADLS | | G-2  G-3  G-4 | 48.56 (32.85)  48.57 (29.30)  32.85 (17.86) | 47.93  49.50  33.48 | 8.551 | **0.014** |
| Quadriceps Muscle Strength | **Non-affected knee** | G-2  G-3  G-4 | 24.70 (9.90)  21.40 (5.38)  18.40 (9.00) | 58.13  42.83  29.98 | 19.805 | **< 0.001** |
|  | **Affected knee** | G-2  G-3  G-4 | 25.70 (9.10)  21.45 (6.53)  18.40 (6.65) | 51.42  41.15  34.00 | 7.492 | **0.024** |
| Hip Adductor Strength | | G-2  G-3  G-4 | 31.00 (17.65)  23.90 (17.05)  19.700 (17.70) | 49.31  39.65  33.91 | 5.856 | 0.054 |
| VAS: Visual analog scale, FTSST: Five time sit to stand test, TUG: Timed up and go test, ICOAP: Intermittent and Constant Osteoarthritis Pain Scale, ISI: Insomnia Severity Index, KOS-ADLS: Knee Outcome Survey-Activities of Daily Living Scale, G-2: Kellgren-Lawrence Grade 2 osteoarthritis, G-3: Kellgren-Lawrence Grade 3 osteoarthritis, G-4: Kellgren-Lawrence Grade 4 osteoarthritis, IQR: Interquartile Range, X^2^: Chi Square, Kruskal-Wallis Test, p < 0.05 means statistical significance. | | | | | | |

| Supplementary Material 2. Pairwise comparisons between groups | | | | | | |
| --- | --- | --- | --- | --- | --- | --- |
|  | | | Pairs | Test Statistics | Std. Error | p-Value |
| VAS | | | G2-G3 | -20.800 | 7.522 | **0.017** |
|  |  |  | G2-G4 | -21.089 | 6.462 | **0.003** |
|  |  |  | G3-G4 | − .289 | 6.462 | 1.000 |
| ICOAP | | **Constant pain score** | G2-G3 | -12.150 | 6.856 | 0.229 |
|  |  |  | G2-G4 | -15.706 | 5.913 | **0.024** |
|  |  |  | G3-G4 | -3.556 | 5.913 | 1.000 |
|  |  | **Total score** | G2-G3 | -12.825 | 7.432 | 0.100 |
|  |  |  | G2-G4 | -21.124 | 6.410 | **0.003** |
|  |  |  | G3-G4 | -5.299 | 6.410 | 1.000 |
| FTSST | | | G2-G3 | -3.025 | 7.531 | 1.000 |
|  |  |  | G2-G4 | -19.304 | 6.470 | **0.009** |
|  |  |  | G3-G4 | -16.279 | 6.470 | **0.036** |
| TUG | | | G2-G3 | -5.975 | 7.530 | 1.000 |
|  |  |  | G2-G4 | -32.542 | 6.470 | **< 0.001** |
|  |  |  | G3-G4 | -26.567 | 6.470 | **< 0.001** |
| KOS-ADLS | | | G2-G3 | 14.449 | 6.412 | 0.073 |
|  |  |  | G2-G4 | 16.024 | 6.412 | **0.037** |
|  |  |  | G3-G4 | -1.575 | 7.434 | 1.000 |
| Quadriceps Muscle Strength | **Affected Knee** | | G2-G3 | 12.850 | 6.283 | 0.123 |
|  |  |  | G2-G4 | 28.157 | 4.404 | **< 0.001** |
|  |  |  | G3-G4 | 15.307 | 7.350 | 0.112 |
|  | **Non-affected Knee** | | G2-G3 | 7.150 | 6.284 | 0.766 |
|  |  |  | G2-G4 | 17.421 | 6.393 | **0.019** |
|  |  |  | G3-G4 | 10.271 | 7.351 | 0.487 |
| VAS: Visual analog scale, FTSST: Five time sit to stand test, TUG: Timed up and go test, ICOAP: Intermittent and Constant Osteoarthritis Pain Scale, KOS-ADLS: Knee Outcome Survey-Activities of Daily Living Scale, G2: Kellgren-Lawrence Grade 2 osteoarthritis, G3: Kellgren-Lawrence Grade 3 osteoarthritis, G4: Kellgren-Lawrence Grade 4 osteoarthritis, IQR: Interquartile Range, Dunn’s post-hoc test with Bonferroni correction following a significant Kruskal-Wallis analysis, p < 0.05 means statistical significance. | | | | | | |
